# Pseudotyped Lentiviral Vectors for Retrograde Gene Delivery into Target Brain Regions

**DOI:** 10.3389/fnana.2017.00065

**Published:** 2017-08-02

**Authors:** Kenta Kobayashi, Ken-ichi Inoue, Soshi Tanabe, Shigeki Kato, Masahiko Takada, Kazuto Kobayashi

**Affiliations:** ^1^Section of Viral Vector Development, National Institute for Physiological Sciences Okazaki, Japan; ^2^SOKENDAI (The Graduate University for Advanced Studies) Hayama, Japan; ^3^Systems Neuroscience Section, Department of Neuroscience, Primate Research Institute, Kyoto University Inuyama, Japan; ^4^Department of Molecular Genetics, Institute of Biomedical Sciences, Fukushima Medical University School of Medicine Fukushima, Japan

**Keywords:** lentiviral vector, fusion envelope glycoprotein, retrograde gene transfer, specific neuronal pathway, gene therapy, Parkinson’s disease

## Abstract

Gene transfer through retrograde axonal transport of viral vectors offers a substantial advantage for analyzing roles of specific neuronal pathways or cell types forming complex neural networks. This genetic approach may also be useful in gene therapy trials by enabling delivery of transgenes into a target brain region distant from the injection site of the vectors. Pseudotyping of a lentiviral vector based on human immunodeficiency virus type 1 (HIV-1) with various fusion envelope glycoproteins composed of different combinations of rabies virus glycoprotein (RV-G) and vesicular stomatitis virus glycoprotein (VSV-G) enhances the efficiency of retrograde gene transfer in both rodent and nonhuman primate brains. The most recently developed lentiviral vector is a pseudotype with fusion glycoprotein type E (FuG-E), which demonstrates highly efficient retrograde gene transfer in the brain. The FuG-E–pseudotyped vector permits powerful experimental strategies for more precisely investigating the mechanisms underlying various brain functions. It also contributes to the development of new gene therapy approaches for neurodegenerative disorders, such as Parkinson’s disease, by delivering genes required for survival and protection into specific neuronal populations. In this review article, we report the properties of the FuG-E–pseudotyped vector, and we describe the application of the vector to neural circuit analysis and the potential use of the FuG-E vector in gene therapy for Parkinson’s disease.

## Introduction

To understand the mechanisms underlying brain functions controlled through complex neural networks, an analysis of the functions of specific neuronal pathways and cell types forming these complex networks is necessary. Recently, we developed a novel lentiviral vector–mediated retrograde gene transfer system that enables analysis of a specific neuronal pathway in the central nervous system (Kato et al., 2013; Kobayashi et al., 2016a, 2017). This system may also be applicable to gene therapy approaches for neurodegenerative disorders by enabling the introduction of transgenes that are involved in neuronal protection and survival into target brain regions.

Retrograde axonal transport of viral vectors offers a substantial advantage for the delivery of a transgene into neuronal populations innervating the injection site of viral vectors (Baumgartner and Shine, 1998; Perrelet et al., 2000; Azzouz et al., 2004; Zheng et al., 2005; Barkats et al., 2006).

**Figure 1:**
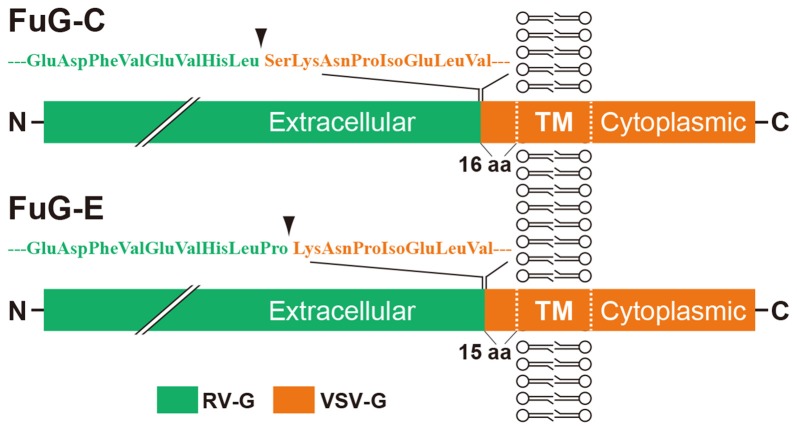
Schematic representations of the structures of fusion glycoprotein types C (FuG-C) and E (FuG-E) used for the NeuRet vector. The extracellular, transmembrane (TM), and cytoplasmic domains are indicated. The amino acid sequences in the membrane proximal region of viral glycoprotein are indicated. Arrowheads show the position of junction between the rabies virus glycoprotein (RV-G) and vesicular stomatitis virus glycoprotein (VSV-G) segments.

Among various kinds of lentiviral vectors, the human immunodeficiency virus type 1 (HIV-1)–based lentiviral vector has been the most commonly used for experimental studies. The HIV-1–based lentiviral vector is a valuable tool for gene therapy trials because it is able to transduce nondividing cells as well as dividing cells, in contrast to other retroviral vectors (Thomas et al., 2003; Kafri, 2004; Wong et al., 2006). The HIV-1-based lentiviral vector possesses a large packaging capacity (8–10 kb; Thomas et al., 2003), and its viral particles are integrated into host genomes after infection (Butler et al., 2002). This vector can be changed by genetically manipulating envelope glycoproteins essential for the vector transduction (reviewed by Cronin et al., 2005; Kobayashi et al., 2017). A conventional type of the HIV-1–based lentiviral vector pseudotyped with vesicular stomatitis virus glycoprotein (VSV-G) has a low efficiency for retrograde gene transfer, which can be improved by pseudotyping with selective variants of rabies virus glycoprotein (RV-G; Mentis et al., 2006; Kato et al., 2007; Federici et al., 2009). To enhance the efficiency of retrograde gene delivery, we previously developed a novel lentiviral vector capable of highly efficient retrograde gene transfer (HiRet) by pseudotyping with fusion glycoprotein type B (FuG-B), which is composed of the extracellular/transmembrane domains of RV-G and the intracellular domain of VSV-G (Kato et al., 2011a,b). Subsequently, we developed another type of lentiviral vector for neuron-specific retrograde gene transfer (NeuRet) with fusion glycoprotein type C (FuG-C), which contains the N-terminal region of the extracellular domain (439 amino acids) of RV-G and the membrane-proximal region of the extracellular domain (16 amino acids) and transmembrane/cytoplasmic domains of VSV-G (Kato et al., 2011c). Figure 1 shows the structure of the glycoprotein. The HiRet and NeuRet vectors exhibit HiRet into various neuronal populations in both mouse and monkey brains (Kato et al., 2011a,c). However, these vectors possess a clear difference in gene transduction property around the injection site; specifically, the HiRet vector efficiently transduces dividing glial and neural stem/progenitor cells, whereas the NeuRet vector scarcely transduces these dividing cells (Kato et al., 2011c). This unique character of the NeuRet vector critically protects against the risk of tumorigenesis caused by altered expression of cellular oncogenes surrounding the insertion site (Li et al., 2002; Hacein-Bey-Abina et al., 2003; Themis et al., 2005; Beard et al., 2007; Marumoto et al., 2009).

Recently, we further developed a novel type of the NeuRet vector with fusion glycoprotein type E (FuG-E) during testing of various glycoproteins, with a shift in the junction between RV-G and VSV-G sequences (Kato et al., 2014). The FuG-E–pseudotyped NeuRet vector displays greater efficiency of retrograde gene transfer in both rodent and nonhuman primate brains compared to the previously developed NeuRet vector. In this review article, we summarize the properties of the FuG-E–pseudotyped NeuRet vector. We also report the application of the FuG-E vector for analyzing the neural circuit mechanism, and we describe the potential use of this vector in gene therapy for neurodegenerative diseases, such as Parkinson’s disease.

## Development of The Neuret Vector Pseudotyped with FuG-E

**Figure 2:**
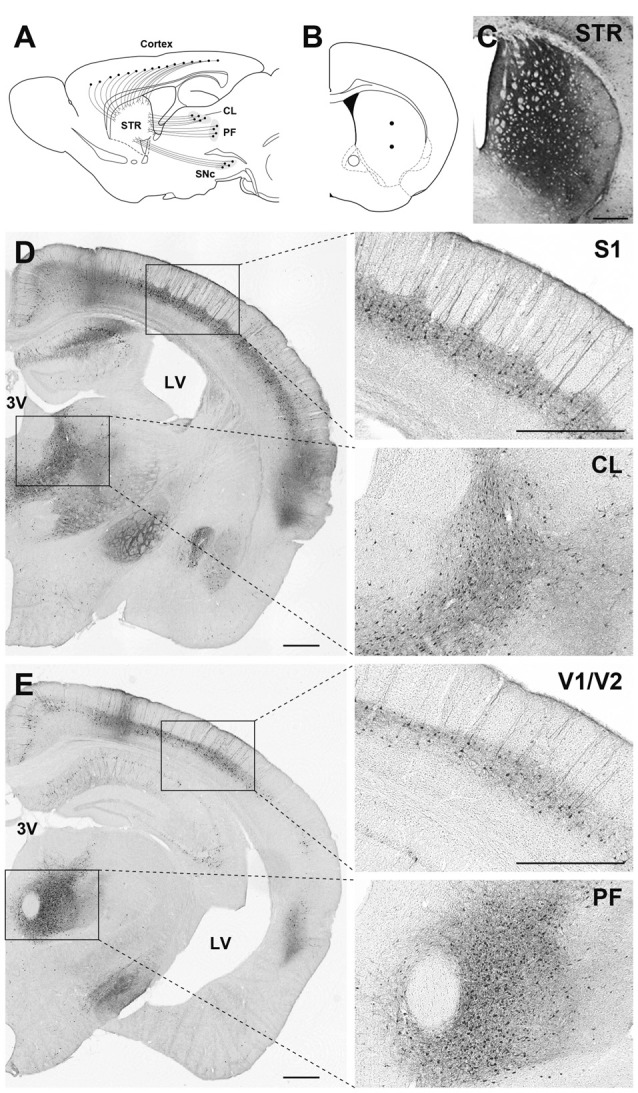
Gene transfer pattern by fusion glycoprotein type E (FuG-E) vector through retrograde gene delivery in the mouse brain. **(A)** Schematic diagram of the projections that innervate the striatum (STR) in mice. CL, central lateral nucleus of the thalamus; PF, parafascicular nucleus of the thalamus; SNc, substantia nigra pars compacta. **(B)** Coordinates for intrastriatal injections. Dots indicate the injection sites. **(C–E)** Immunohistochemical detection of transgene expression. The FuG-E–pseudotyped vector encoding GFP transgene (3.6 × 10^12^ genome copies/ml, 0.75 μl/site for two sites) was injected into the STR in mice, and brain sections through the STR **(C)**, CL** (D)** and PF **(E)** were used for immunohistochemical staining with anti-GFP antibody. Many immunopositive cells were detected in various neuronal populations in cortical areas including the primary somatosensory cortex (S1), primary and secondary visual cortices (V1/V2), and the intralaminar thalamic regions (CL, PF) that innervate the STR. Right images are magnified views of the squares in the left image **(A)** or **(B)**. 3V, third ventricle; LV, lateral ventricle. Scale bar: 500 μm.

Our previous studies suggest that pseudotyping of lentiviral vectors with different types of fusion glycoproteins influences the efficiency of retrograde gene transfer and the transduction property of cells around the injection site (for review see Kobayashi et al., 2017). As observed with FuG-C, fusion in the membrane-proximal region of viral envelope glycoproteins improves the efficiency of retrograde gene transfer and suppresses gene transduction into dividing cells around the injection site (Kato et al., 2011c). However, the position of the junction between RV-G and VSV-G segments in fusion glycoproteins that confers the most efficient gene transfer has been unclear. To optimize the junction of these two glycoproteins for efficient retrograde gene transfer, we produced various types of chimeric glycoproteins, in which the junction between the RV-G and VSV-G segments diverged in their membrane-proximal region, and we tested the *in vivo* gene transfer activity of the pseudotyped vectors (Kato et al., 2014). First, we screened the pseudotyped vectors based on the efficiency of gene delivery into thalamic neurons after intrastriatal injection in mice. We found that the vector pseudotyped with FuG-E, comprising the N-terminal region of the extracellular domain (440 amino acids) of RV-G and the membrane-proximal region of the extracellular domain (15 amino acids) and transmembrane/cytoplasmic domains of VSV-G (see Figure 1 for the structure of glycoprotein), displayed the most efficient retrograde gene transfer into thalamostriatal neurons (i.e., a 1.2-fold greater transduction compared with the original FuG-C vector, based on data from Kato et al., 2014). The FuG-E vector also showed a high level of retrograde gene delivery into corticostriatal neurons in different cortical areas of the mouse brain, with a similar extent of gene transduction into thalamostriatal neurons. In addition, this vector pseudotype retained the property of neuron-specific gene transduction around the injection site. The typical expression pattern of the FuG-E vector through retrograde gene transfer in the mouse brain is shown in Figure 2.

## Gene Delivery of FuG-E–Pseudotyped Vector into Nonhuman Primate Brain

**Figure 3:**
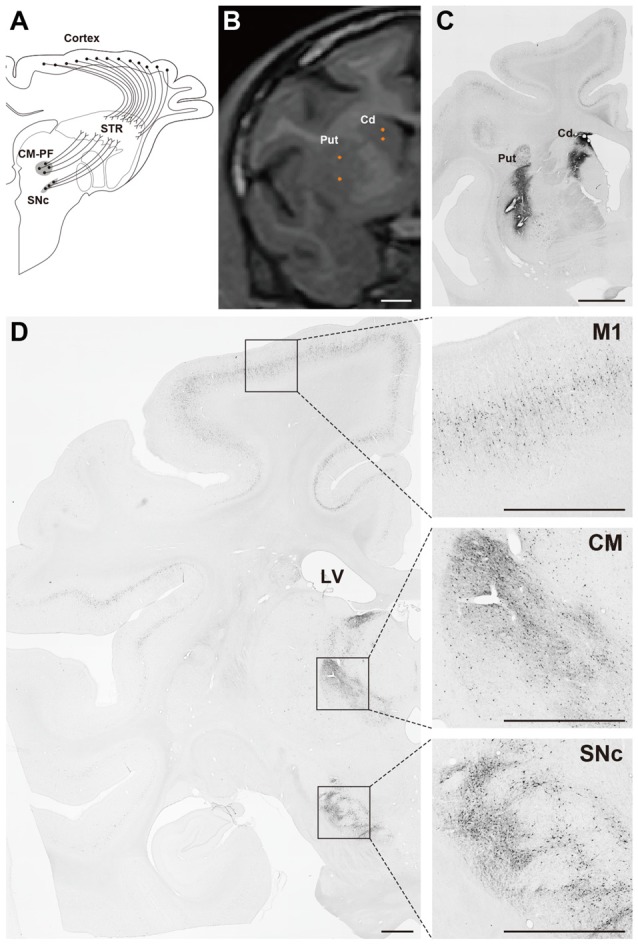
Gene transfer pattern by FuG-E vector via retrograde gene delivery in the monkey brain. **(A)** Schematic diagram of the projections that innervate the striatum (STR) in macaque monkeys. CM-PF, centromedian -parafascicular complex of the thalamus; SNc, substantia nigra pars compacta. **(B)** Coordinates for intrastriatal injections. Representative injection sites in the caudate nucleus (Cd) and putamen (Put) are shown in a magnetic resonance image. **(C,D)** Immunohistochemical detection of transgene expression. The FuG-E–pseudotyped vector encoding GFP transgene (7.0 × 10^10^ genome copies/ml, 3–5 μl/ site × 16 sites) was injected into the Cd and Put in a macaque monkey and brain sections through the STR **(C)** and other brain regions **(D)** were immunostained with anti-GFP antibody. A number of immunopositive cells were detected in various neuronal populations in cortical areas including the primary motor cortex (M1), thalamic (CM), and nigral (SNc) regions innervating the STR. Right images are magnified views of the squares in the left image. LV, lateral ventricle. Scale bar: 2 mm.

The gene transfer pattern of the FuG-E–pseudotyped vector was investigated in the nonhuman primate brain (Tanabe et al., 2017). The FuG-E pseudotype was injected into the striatum of macaque monkeys, and the efficiency of retrograde gene transfer was compared to that of the parental FuG-C–pseudotyped vector. The FuG-E vector exhibited greater efficiency of retrograde gene transfer into various neuronal populations in subcortical regions innervating the striatum than the FuG-C vector; specifically, 1.2-, 2.0- and 1.4-fold elevation of transfer efficiency into the substantia nigra pars compacta, centromedian-parafascicular nuclear complex of the thalamus, and centrolateral nucleus of the thalamus, respectively (data from Tanabe et al., 2017). In cortical areas, the gene transfer efficiency of the FuG-E vector in the hemisphere ipsilateral to the vector-injected side was 2.2-, 2.1-, 2.1- and 1.7-fold higher in the primary motor area, supplementary motor area, Brodmann’s area 8b and Brodmann’s area 46d, respectively as compared with that of the FuG-C vector, and the efficiency of the FuG-E vector in the contralateral hemisphere was increased to 5.8-, 4.1-, 8.7- and 5.9-fold of the value of the FuG-C vector in the respective four areas (data from Tanabe et al., 2017). Moreover, the FuG-E vector maintained the property of neuron-specific gene transduction around the injection site. The representative gene expression pattern of the FuG-E vector via retrograde gene transfer in the monkey brain is demonstrated in Figure 3.

These analyses highlight that the FuG-E–pseudotyped vector achieves retrograde gene delivery with greater efficiency compared with the NeuRet vector pseudotyped with FuG-C, suggesting the important role of the sequences of the membrane-proximal regions of viral envelope glycoproteins in retrograde gene delivery. This type of vector therefore provides a powerful experimental strategy to investigate the mechanisms underlying various neural functions more precisely, and it offers a promising genetic tool for gene therapy trials for neurological and neurodegenerative disorders.

## Other Viral Vectors for Retrograde Gene Transfer

Rabies virus (RV) is well known to spread transsynaptically in the retrograde direction in the central nervous system (Ugolini, 2008). A recombinant glycoprotein-deleted RV (RVΔG) vector has widely been used for network-tracing studies (Callaway, 2008; Callaway and Luo, 2015). The RVΔG vector pseudotyped with the envelope protein of subgroup A avian sarcoma and leukosis virus (EnvA) can specifically infect neurons that have been genetically targeted for expression of the EnvA receptor (TVA). The *trans*-complementation of the RVΔG vector with RV-G in neurons expressing TVA allows the vector to move monosynaptically to neuronal populations projecting to the TVA-expressing neurons (Wickersham et al., 2007; Miyamichi et al., 2011; Beier et al., 2015; Schwarz et al., 2015). This system is commonly used to identify the direct monosynaptic input to genetically defined neurons. Moreover, a pseudorabies virus (PRV) vector (Pomeranz et al., 2005; Braz et al., 2009; Ohara et al., 2009; Oyibo et al., 2014), a herpes simplex virus type 1 vector (Zemanick et al., 1991; LaVail et al., 1997; Fenno et al., 2014; Gremel et al., 2016) and a canine adenovirus serotype 2 vector (Bru et al., 2010; Senn et al., 2014) can also be transported retrogradely through axons. Among the viral vectors already mentioned, the RV and PRV vectors have often been used for short-term experimental studies such as retrograde neuron labeling. These vectors, like the lentiviral vector, can be handled under biosafety level 2 conditions. However, neither the RV nor the PRV vector are necessarily suitable for prolonged physiological studies because of cytotoxicity; the lentiviral vector does not share this characteristic. Properly detoxified viral vectors would be useful for various physiological applications over a long period of time.

Some serotypes of AAV vectors transfer foreign genes in a retrograde fashion in the central nervous system (Masamizu et al., 2011; Aschauer et al., 2013; Löw et al., 2013; Salegio et al., 2013; San Sebastian et al., 2013). In addition, a new AAV variant vector, termed rAAV2-retro vector, has recently been shown to robustly induce transgene expression in the retrograde direction (Tervo et al., 2016). In the future, stringent comparison of the efficiency of retrograde gene transfer of the FuG-E vector and these AAV vectors will be necessary in various neuronal pathways of diverse animal models. A major drawback of the AAV vector is that the packaging size of the transgene is limited, and large genes are therefore not currently suitable for use. The lentiviral vector can convey a larger transgene (8–10 kb) than the AAV vector (5 kb; Thomas et al., 2003), so the FuG-E vector enables use of more genes of interest than the AAV vector. If the AAV vector could be modified to carry a larger transgene, it would provide a more powerful experimental tool for analysis of brain functions. Also, the AAV vector can be handled under biosafety level 1 conditions, while the lentiviral vector is a biosafety level 2 organism; consequently, the AAV vector is more tractable than the lentiviral vector. In addition, AAV vector particles are physiochemically more stable and can be preserved for a far longer period than lentiviral vector particles. A more durable lentiviral vector with a high efficiency of retrograde gene transfer would offer an extremely appealing experimental tool.

## Application of FuG-E Vector to Neural Circuit Analysis

The FuG-E–pseudotyped vector has provided a useful gene engineering tool for experimental studies of neural circuit functions (for review see Kobayashi et al., 2017). For instance, the neural circuit mechanism underlying motor function recovery following stroke was investigated by selectively and reversibly blocking the specific neuronal pathway (Ishida et al., 2016). When synaptic transmission in the neuronal pathway projecting from the ipsilesional motor cortex to the ipsilesional red nucleus was reversibly blocked, rehabilitated rats displayed a remarkable decrease in motor performance, indicating an essential role of the ipsilateral cortico–red nucleus pathway in motor function recovery through rehabilitation (Ishida et al., 2016). The role of propriospinal neuron–mediated neuronal pathway in the recovery of hand dexterity after corticospinal tract lesions was examined by the same synaptic blockade procedure (Tohyama et al., 2017). In monkeys, recovery of dexterous hand movements after corticospinal tract lesions was perturbed by reversibly blocking the synaptic transmission from propriospinal neurons to spinal motor neurons, showing an important role of the propriospinal neuron–spinal motor neuron pathway in promoting the recovery of hand dexterity after the lesion. When the activity of dopaminergic pathway from the ventral tegmental area to the nucleus accumbens was specifically suppressed by a chemogenetic approach in mice under a neuropathic pain-like state, exercise-induced hypoalgesia was markedly impaired (Wakaizumi et al., 2016). These results indicate that the ventral tegmental area–nucleus accumbens pathway is involved in the anti-nociception effect caused by exercise under a neuropathic pain-like state. In a recent report, we investigated the role of a small GTPase Rho signaling pathway mediated by Rho-kinase, one of the major target molecules of Rho, in corticostriatal neuron survival (Kobayashi et al., 2016b). Conditional blockade of the Rho/Rho-kinase signaling pathway in mouse corticostriatal neurons caused a marked decrease in the number of these neurons, which was attributed to enhanced apoptosis (Kobayashi et al., 2016b). These data show that the Rho/Rho-kinase signaling pathway is essential for the survival of corticostriatal neurons. In addition, the FuG-E vector is certainly applicable to other experimental strategies, such as selective pathway targeting (Kato et al., 2011b; Inoue et al., 2012) and optogenetic (Matsuda et al., 2017) techniques.

## Potential Use of FuG-E Vector in Gene Therapy Trials for Parkinson’s Disease

Parkinson’s disease is a neurodegenerative disorder characterized by progressive loss of nigrostriatal dopaminergic neurons; the loss of these neurons induces motor dysfunctions (Fahn, 2003; Obeso et al., 2010). Various gene therapy trials targeting dopaminergic neurons have been carried out using a rodent or a nonhuman primate model of Parkinson’s disease (Kim et al., 2012; Redmond et al., 2013; Ren et al., 2013; Oh et al., 2015). However, gene transfer specifically into nigrostriatal dopaminergic neurons has not yet been achieved. Gene transfer into specific neuronal populations enables assessment of the clinical effects of the transgene without the effect of nonspecific transgene actions in other cell populations. The FuG-E vector achieves a higher efficiency of retrograde gene delivery compared with the previously developed NeuRet vector, and it permits neuron-specific gene transduction around the injection site in the brain (Kato et al., 2014). Interestingly, retrograde gene transfer of the FuG-E vector into mouse nigrostriatal dopaminergic neurons is less efficient, whereas the FuG-E vector displays remarkably high transduction efficiency into the dopaminergic neurons in monkeys (Tanabe et al., 2017). In the near future, our newly developed FuG-E vector system with these attractive characteristics may serve as a powerful genetic tool in gene therapy trials for Parkinson’s disease.

## Conclusion

Gene transduction via viral vectors offers great advantages in genetic manipulation of neural circuit functions in diverse animal models, including rodents and nonhuman primates. We have developed novel types of lentiviral vectors, the HiRet and NeuRet vectors, which display HiRet in the central nervous system. These viral vectors enable analysis of the roles of specific neuronal pathways or signaling molecules with critical functions in complex neural circuits. Viral vector–mediated retrograde gene delivery is a particularly important genetic technology in nonhuman primates because conventional transgenic approaches generally used for mice are hardly applicable to monkeys. Notably, the NeuRet vector possesses a unique characteristic in that it scarcely transduces dividing glial and neural stem/progenitor cells around the injection site. This property of the NeuRet vector is essential for genetic treatment of neurodegenerative diseases because viral vector integration into dividing cells may increase the risk of tumorigenesis in the brain. The NeuRet vector permits reducing the risk of tumorigenesis by suppressing gene delivery into glial and neural stem/progenitor cells. The NeuRet vector exhibits efficient retrograde gene transfer into the nigrostriatal pathway in primates, and the FuG-E vector has remarkably higher gene transfer efficiency in this pathway than the FuG-C vector. The FuG-E vector possessing these advantageous characteristics could contribute to the development of novel primate models for Parkinson’s disease. Furthermore, the FuG-E vector may provide promising approaches to gene therapy for Parkinson’s disease by delivering genes required for neuronal survival and protection into nigrostriatal dopaminergic neurons.

## Ethics Statement

All experiments were performed in accordance with the guidelines of the National Institutes of Health, and were approved by the Animal Research Committee of Fukushima Medical University for rodents and the Animal Welfare and Animal Care Committee of the Primate Research Institute, Kyoto University for monkeys. We made all efforts to minimize the number of animals used and their suffering.

## Author Contributions

KeK wrote the present manuscript; KI, ST and SK designed figures; MT and KaK critically read and edited the manuscript.

## Conflict of Interest Statement

The authors declare that the research was conducted in the absence of any commercial or financial relationships that could be construed as a potential conflict of interest.
